# Lamotrigine Reduces Inflammatory Response and Ameliorates Executive Function Deterioration in an Alzheimer's-Like Mouse Model

**DOI:** 10.1155/2016/7810196

**Published:** 2016-11-30

**Authors:** Kexin Wang, Alejandro Fernandez-Escobar, Shuhong Han, Ping Zhu, Jun-Hui Wang, Yu Sun

**Affiliations:** ^1^Department of General Surgery, Qilu Hospital of Shandong University, Jinan, Shandong 250012, China; ^2^School of Medicine, CES University, Medellin, Antioquia, Colombia; ^3^Department of Medicine, Division of Rheumatology & Clinical Immunology, University of Florida, Gainesville, FL 32610-0275, USA; ^4^Department of Ophthalmology, College of Medicine, University of Florida, Gainesville, FL 32610-0284, USA; ^5^Department of Physiology, University of Toronto, Toronto, ON, Canada M5S1A8; ^6^Department of Endocrinology, Qilu Hospital of Shandong University, Jinan, Shandong 250012, China

## Abstract

Alzheimer's disease (AD) has been described in the literature, to be associated with impairment of executive function which develops early in the course of disease, and an effective treatment for this clinical feature remains elusive. Preclinical studies have implied that lamotrigine, an antiepileptic agent, could be a potential treatment for executive dysfunction in AD patients. Although there have been promising results in previous studies with lamotrigine, executive function has never been measured using animal models. The aim of the present study was to evaluate the effects of lamotrigine on executive function and determine whether lamotrigine can attenuate inflammatory response in an AD mouse model. Nontransgenic and transgenic mice were treated with lamotrigine (0 or 30 mg/kg/day) in a standard laboratory chow diet starting at 3 months of age. After 6 months of continuous lamotrigine administration, there was a marked improvement in executive function and a significant attenuation in the expression of proinflammatory cytokines. These results suggest that lamotrigine could ameliorate executive dysfunction and brain inflammatory response in the mouse model of AD and early lamotrigine intervention may be a promising therapeutic strategy for AD.

## 1. Introduction

AD is the most common dementia in elderly population, which is characterized by the extracellular deposition of *β*-amyloid (A*β*) peptide in senile plaques, intracellular neurofibrillary tangles, and synaptic deterioration in the central nervous system (CNS) [1]. AD is defined as a progressive disease that destroys memory and other important mental functions, including executive dysfunction in the early stage of the disease [2]. Executive function encompasses a number of cognitive abilities such as working memory, cognitive flexibility, inhibitory control, and another complex functions as planning, problem solving, and abstract reasoning [2]. Examples of executive dysfunction in AD patients include poor selective and divided attention, failed inhibition of interfering stimuli, and poor manipulation skills [3].

Currently, therapeutic agents can only moderately improve the symptoms of AD and no disease-modifying agent has been identified [4]. Lamotrigine (LTG) is a new anticonvulsant drug for the treatment of partial and secondarily generalized seizures [5]. LTG may also be effective in the management of bipolar disorder and improvement of mood disorders [6]. More importantly, LTG has been found to effectively attenuate some behavioral abnormalities, including cognitive impairment in AD patients and animal models [7, 8]. However, the effect of LTG on executive dysfunction in AD has never been measured. Systemic inflammation has been found to impair prefrontal cortex function by in aged animals [9, 10]. Since executive function has traditionally been localized to the prefrontal cortex, several inflammatory biomarkers are validated to be adversely associated cognitive function in a clinical study on human subjects [10].

Although the actual mechanisms involved in AD are not very clear, a large body of evidence implicates neuroinflammatory processes in the etiology and progression of AD [11]. Inflammation is an early feature of AD pathophysiology [12]. Among the proinflammatory cytokines, interleukin 6 (IL-6) and interleukin 1 beta (IL-1*β*) have a number of functions that are relevant to AD, such as excessive expression of A*β* precursor protein and other plaque-associated proteins, and induction of astrocyte activation and astrocytic overexpression of S100B [13, 14]. In a 12-month-old Tg2576 AD mice, IL-1*β* mRNA level is significantly increased and it is associated with severe cognitive dysfunction which could be attenuated by genetically overexpression of IL-1 receptor antagonist [11]. In AD patients, IL-6 is detected in plaques of AD patients prior to the onset of neuritic degeneration [15]. These findings suggest that these two cytokines might be good therapeutic targets for treatment of AD. Literature has suggested that lamotrigine attenuated the hyperalgesia in an animal model of inflammatory pain [16].

The goals of this study were to examine the effects of LTG on the executive dysfunction and IL-1*β* changes in an APP/PS1 mouse model. For the first time, we found that LTG could effectively attenuate executive dysfunction in AD mice, along with an inhibitory effect on the production of IL-1*β*. With these novel findings, our study provided new evidence that the anticonvulsant drug, LTG, may prevent the deterioration of executive function in AD mice by influencing the production of cytokines, such as IL-1*β* and IL-6.

## 2. Materials and Methods

### 2.1. Animals and Drug Treatment

3-month-old female APP/PS1 mice and age-matched and gender-matched wild-type (WT) mice were used in the present study. The animals were housed at a 12 h day/12 h night light cycle with* ad libitum* access to water and food, under controlled laboratory conditions. All procedures were performed in accordance with the guidelines set by the Canadian Council on Animal Care and approved by the University Committee on Animal Care and Supply and the University of Manitoba Animal Care Committee. LTG (Sigma-Aldrich) were prepared in a diet of standard laboratory chow (30 mg/kg per day) as previous report [8]. There are four different groups in this study: wild-type (WT); WT plus LTG; APP/PS1; APP/PS1 plus LTG. The mice began to receive LTG administration (target dose: 30 mg/kg/day) from the age of 3 months. Mice were treated with LTG or water for 6 months until they became 9 months old. Animals were weighed every other week to monitor body weight changes (see Supplementary Fig. 1 in Supplementary Material available online at http://dx.doi.org/10.1155/2016/7810196). Control APP/PS1 and WT mice received the same chow without LTG. The target dose chosen here was based on the previous report [8].

### 2.2. Primary Cultures of Astrocyte

Cerebral cortical astrocytes were prepared from one-day-old littermates of APP/PS1 mice as previously described with minor modifications [17]. Cells were incubated in a 95% humidified Napco incubator (Precision Scientific Inc., IL, USA) at 37°C with 5% CO_2_. The astrocyte cultures were fed Dulbecco's Modified Eagle's Medium (DMEM, Life Technologies) with 10% fetal bovine serum (FBS, Life Technologies) every 3 days until 4 weeks when the astrocytes became functionally mature. 4-week-old astrocyte cultures were used for immunofluorescence and biochemistry studies.

### 2.3. Puzzle Box Test

A puzzle box assay, adapted from Milenkovic et al. [18], was used to assess mouse executive function. The apparatus is a Plexiglas white box, divided by a removable barrier into a goal box (goal zone, 15 × 28 cm) and a larger start box (start zone, 58 × 28 cm) with an underpass that allows animals to move between the two compartments. Mice were placed in the start box and then the latency that mice enter the goal box was recorded. Mice underwent a total of nine trials (T1–T9) over 3 consecutive days with increasingly difficult puzzles to solve between the start and goal box as previously reported [19]. Mice first use an open doorway (T1), and then the doorway is blocked and an underpass is used to allow the mice reach the goal box (T2). This challenge is repeated in T3 to assess short-term learning and memory of the task. On the second day of testing, the challenge of trial 3 is repeated again to measure short-term learning and memory of the same task (T4). On T5, the underpass is blocked with bedding and mice need to burrow the bedding to reach the goal box. This challenge is also repeated in T6 (second day) and T7 (third day) to assess short- or long-term memory of this task. The underpass is blocked with cardboard plug on T8 and T9 of the third day and mice need to move the plug out of the underpass before they enter the goal box. A 2-min interval was maintained for trials within a given day. A maximum time of 5 min was allowed for completion of each trial.

### 2.4. Immunofluorescence Staining

Cell immunofluorescence was performed at room temperature. The cells were fixed with 4% paraformaldehyde for 30 min. After washing twice with PBS, the cells were permeabilized with 0.2% Triton X-100 for 30 min and then blocked with 3% bovine serum albumin for 2 hrs. After an overnight incubation with primary antibody to glial fibrillary acidic protein (GFAP) (1 : 1000, Millipore), the following day the cells were washed three times with PBS and incubated with second antibody of Alexa Fluor 594 (1 : 1,000, Invitrogen-Molecular Probes) for 2 hours. 1 *μ*g/mL Hoechst 33342 (Invitrogen-Molecular Probes) was used for nuclear staining.

### 2.5. Western Blot

Brain tissues from frontal cortex of mice were lysed in a cold lysis buffer containing 1% protease inhibitor cocktail (Sigma). The extracted proteins were separated by electrophoresis with the 12% SDS-PAGE and transferred onto nitrocellulose membranes. The target proteins were measured using the primary antibodies of anti-IL-1 (1 : 1000, Santa Cruz) or GFAP (1 : 1000, Santa Cruz) and the corresponding secondary antibody, followed by development with an ECL kit (PerkinElmer). The antibodies against *β*-actin (1 : 4000, Santa Cruz) or GAPDH (1 : 4000, Abcam) were used here as an internal control. Quantitative results are expressed as a ratio of target proteins to their internal controls accordingly.

### 2.6. Immunohistochemistry and Amyloid Plaque Staining

Coronal brain sections (30 *μ*m) were stained with the ABC Peroxidase Staining Kit (Pierce) by following the manufacture's protocol. All sections were blocked by 3% hydrogen peroxide in PBS for 20 minutes. Sections were incubated at room temperature for 30 minutes with 5% serum and 0.3% Triton X-100 in PBS and then incubated overnight at 4°C with GFAP antibody (1 : 4,000; AB5541; Millipore). Secondary biotinylated antibody was used at a dilution of 1 : 1000 (Vector Laboratories). After washing 3 times with PBST, sections were immersed into ABC reagent for 30 min. After the GFAP staining, amyloid plaques were detected with commercial kit of Congo Red (Sigma-Aldrich) by following the instruction. Sections were immersed in Mayer's Hematoxylin Solution for 5 min and then rinsed in tap water for 5 min. After being incubated in Alkaline Sodium Chloride Solution for 20 min, the sections were placed in filtered Alkaline Congo Red Solution for another 20 min. All sections were rinsed with absolute ethanol for 3 times and cleared in xylene before being mounted.

### 2.7. Enzyme Linked Immunosorbent Assay (ELISA)

The concentrations of IL-6 and IL-1*β* in brain tissue and astrocytic cell lysate were measured by using a commercial ELISA kit (eBioscience), following the manufacturer's protocol. Each sample was assayed in duplicate at appropriate dilutions so that relative luminescent units fell within the linear range of standard curves. The value of IL-6 and IL-1*β* from each well was normalized and expressed as a ratio to total loading protein.

### 2.8. Statistical Analysis

All data was analyzed using GraphPad PRISM 5.0 software (GraphPad Software). Comparison between groups was performed using analysis of one-way ANOVA followed by Newman–Keuls post hoc test or Student's *t*-test. Data are shown as mean ± SEM. Differences were considered statistically significant at *p* < 0.05.

## 3. Results

### 3.1. Amyloid Plaque Deposition and Astrocyte Activation in Brains of APP/PS1 Mice

**Figure 1:**
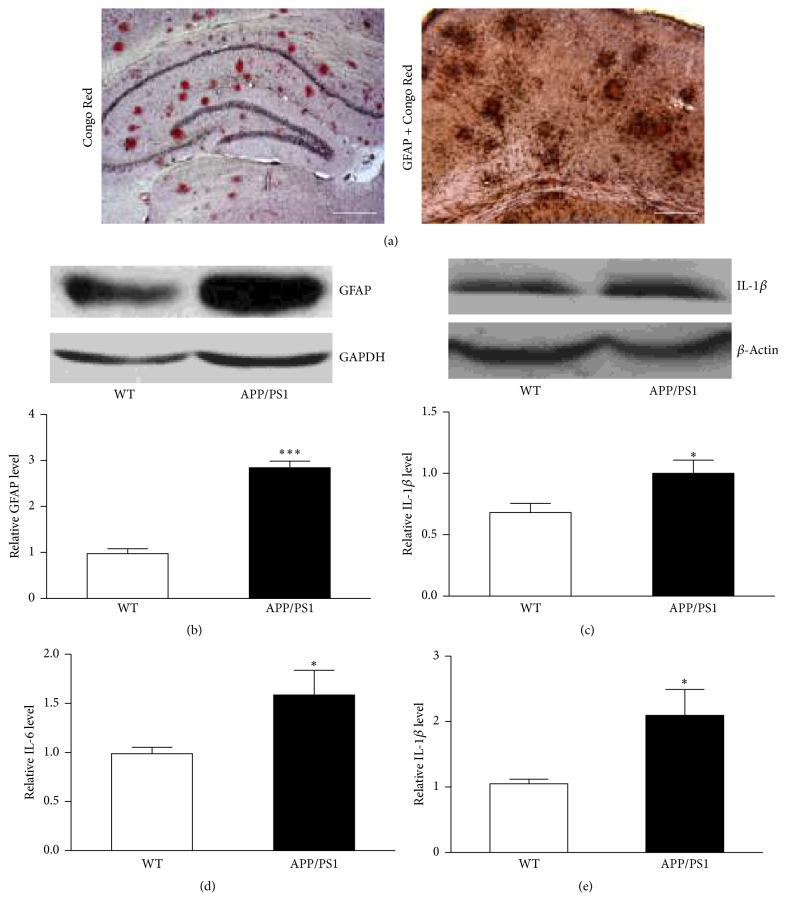
APP/PS1 mice (9 months old) showed plaque deposition, astrocyte activation, and higher level of IL-6 and IL-1*β* production in the brains. (a) Representative photograph of plaque deposition (Congo Red) in 9-month-old APP/PS1 mice (left); representative photograph of costaining of GFAP and Congo Red in 9-month-old APP/PS1 mice (right). Scale bar represents 500 *μ*m. (b) Western blot analysis showed an increase of GFAP expression in APP/PS1 mouse brain cortex (^*∗∗∗*^ *p* < 0.001). *n* = 4. (c) Western blot analysis showed an increase of IL-1*β* in APP/PS1 mouse brain cortex (^*∗*^ *p* < 0.05). *n* = 4. (d) IL-6 was measured with ELISA kit in the brain tissue of cortical area of WT and APP/PS1 mice (^*∗*^ *p* < 0.05). *n* = 5. (e) IL-1 *β* was measured with ELISA kit in the brain tissue of cortical area of WT and APP/PS1 mice (^*∗*^ *p* < 0.05). *n* = 5. All data are expressed as means ± SEM.

Amyloid deposition and accumulation are well-documented pathological alterations observed in brains of AD patients and some AD animal models [20, 21]. In the present study, we found obvious A*β* deposits in mouse brain of 9 months old with Congo Red staining (Figure 1(a)) and astrocyte activation with immunohistochemistry (Figure 1(a)). As shown in Figure 1(a), costaining of GFAP and plaques showed activated astrocytes accumulated as clusters surrounding red plaques. Comparison of WT mice of these two types of staining were presented as Supplementary Fig. 2 and Fig. 3. Consistent with immunohistochemistry finding, a significant upregulation of GFAP protein expression in AD mouse was further confirmed by western blot analysis (Figure 1(b)). We also tested whether IL-1*β* was overexpressed in frontal cortexes of these AD mice. As shown in Figures 1(c) and 1(e), western blot and ELISA analysis demonstrated notable increase of IL-1*β* level in the brain tissues of APP/PS1 mice. IL-6 protein level was assessed with ELISA kit and our results show an increase of this cytokine in APP/PS1 mice as well (Figure 1(d)). The above results implied that activated astrocytes in APP/PS1 mice boosted the generation of IL-6 and IL-1*β* proteins.

### 3.2. Lamotrigine Inhibits Inflammatory Response of APP/PS1 Mice and Cultured Astrocytes

**Figure 2:**
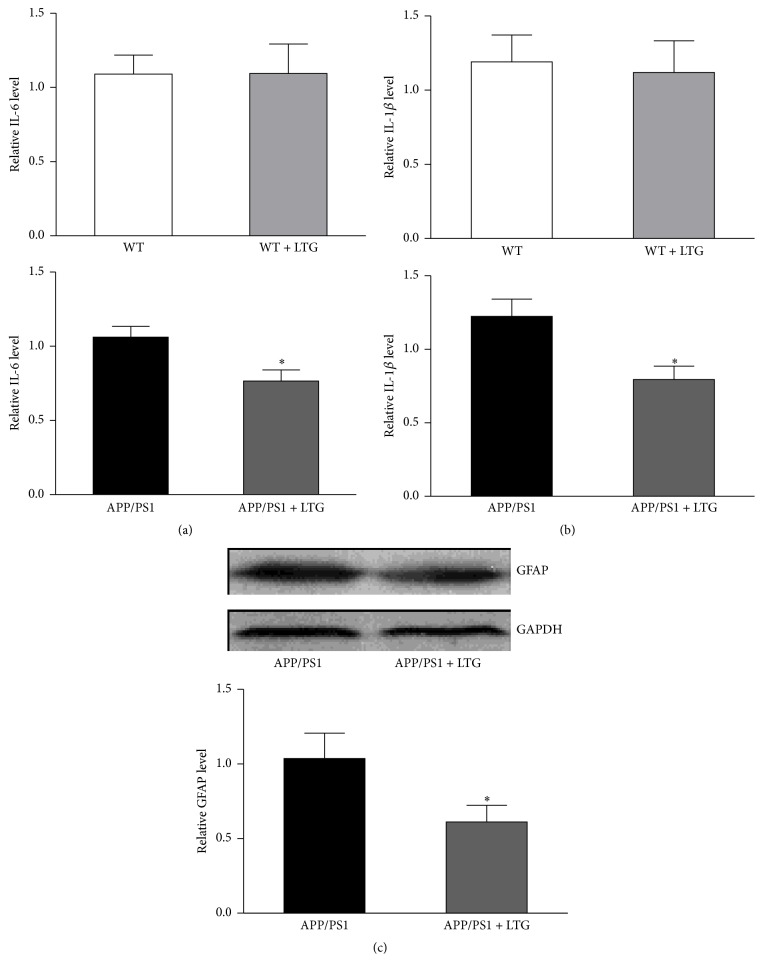
Lamotrigine decreased IL-6 and IL-1*β* production and inhibited GAFP expression in brain cortex of APP/PS1 mice. (a) IL-6 was measured with ELISA kit in the brain tissue of cortical area of WT and APP/PS1 mice with or without lamotrigine treatment (^*∗*^ *p* < 0.05). *n* = 5. (b) IL-1*β* was measured with ELISA kit in the brain tissue of cortical area of WT and APP/PS1 mice with or without lamotrigine treatment (^*∗*^ *p* < 0.05). *n* = 5. (c) GFAP expression in APP/PS1 mouse brain cortex was measured with western blot analysis in the brain tissue of cortical area of APP/PS1 mice with or without lamotrigine treatment (^*∗*^ *p* < 0.05). *n* = 4. All data are expressed as means ± SEM.

**Figure 3:**
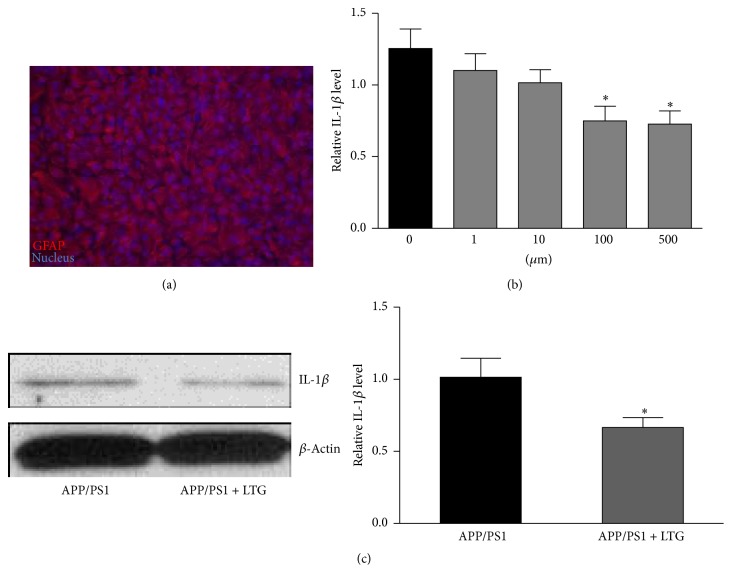
Lamotrigine inhibits IL-1*β* production of cultured astrocytes from APP/PS1 mice. (a) Representative photograph of 4-week-old cultures astrocytes labeled with the specific marker protein of GFAP and Hochest 33258 for nucleus staining. (b) IL-1*β* was measured with ELISA kit in the cultured astrocytes treated with lamotrigine for 24 hours at different doses (^*∗*^ *p* < 0.05). *n* = 14–17. (c) IL-1*β* expression level was measured with western blot analysis in cultured astrocyte treated with 500 *μ*M lamotrigine for 24 hours (^*∗*^ *p* < 0.05). *n* = 5. All data are expressed as means ± SEM.

Next, we tested whether LTG could reduce the IL-6 and IL-1*β* protein level in the APP/PS1 mice. We performed ELISA analysis on the tissue lysate of frontal cortex. As shown in Figures 2(a) and 2(b), LTG treatment significantly reduced the expression level of IL-6 and IL-1*β* in the brain tissues of APP/PS1 mice but had no effects on the WT mice. We also asked whether LTG could affect the astrocyte activation in AD mice. Western blot analysis was performed and our results revealed that LTG significantly suppressed the overexpression of GFAP in brain cortex of AD mice (Figure 2(c)), which suggested an inhibitory effect on astrocyte function. To investigate the capacity of astrocyte in producing IL-1*β*, we measured the IL-1*β* protein level in the cultured cortical astrocytes from APP/PS1 mice. Primary astrocytes from APP/PS1 littermates (postnatal day 1) were cultured for 4 weeks. The cultures were stained with the GFAP antibody and Hoechst 33342. The results indicated that all cultures have similar number of cells (approximately 7~8 × 10^6^) when becoming confluent (Figure 3(a)), of which more than 95% are GFAP-positive cells. These 4-week-old cultures were treated with LTG on different doses (1, 10, 100, and 500 *μ*M). After 24 hours, cell lysates were collected and IL-1*β* protein level was measured by ELISA kit. As shown in Figure 3(b), LTG (100 and 500 *μ*M) significantly reduced the levels of IL-1*β* in astrocytes. The above reducing effect of LTG on IL-1*β* in APP/PS1 mice was further confirmed by western blot analysis in Figure 3(c). LTG (500 *μ*M) treatment effectively lowered the protein level of IL-1*β* of astrocytes after 24-hour treatment.

### 3.3. Lamotrigine Attenuates Executive Function Impairment but Not Memory Deficits of APP/PS1 Mice in a Puzzle Box Test

**Figure 4:**
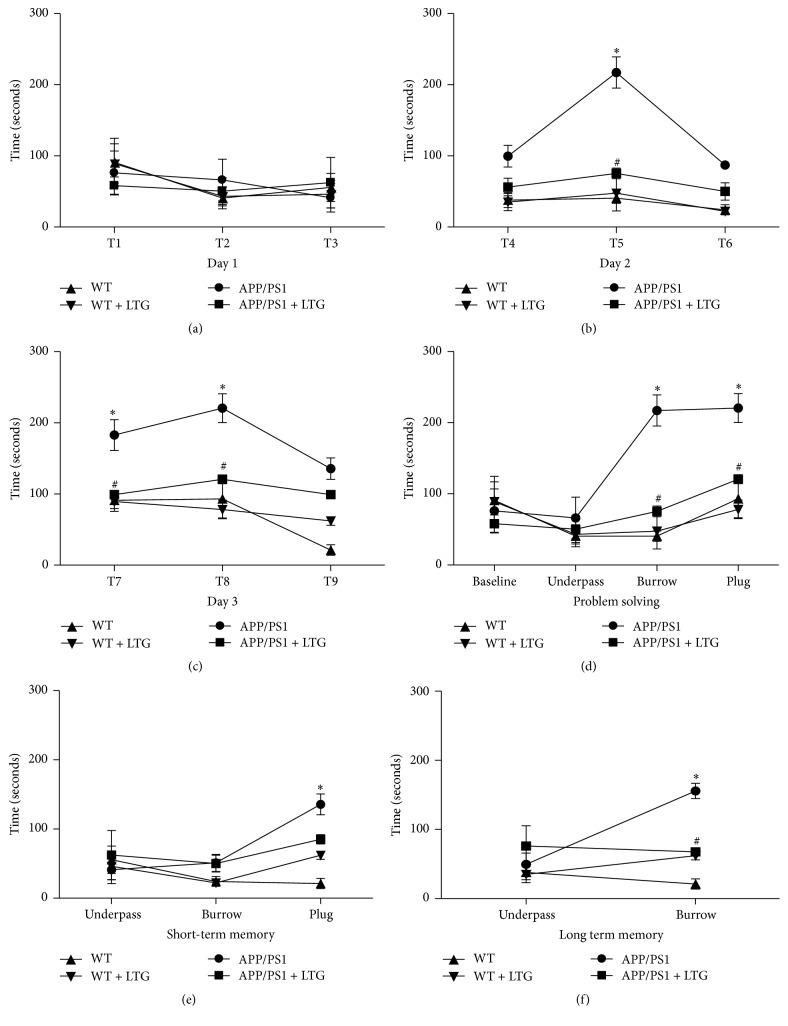
Lamotrigine attenuates executive function deficits of APP/PS1 mice in a puzzle box test. (a) Latencies of mice in each group to complete the task on day 1 (T1–T3). (b) Latencies of mice in each group to complete the task on day 1 (T4–T6). (c) Latencies of mice in each group to complete the task on day 1 (T7–T9). (d) Latencies of mice in each group to solve a new problem during the 3-day test. (e) Latencies of mice in each group to solve a repeat new problem after 3-minute interval during the 3-day test (short-term memory). (f) Latencies of mice in each group to solve a repeat new problem after 24-hour interval during the 3-day test (long-term memory). All data are expressed as means ± SEM. ^*∗*^ *p* < 0.05 versus WT; ^#^ *p* < 0.05 versus APP/PS1, *n* = 8.

Executive function impairment is indicated in the early course of AD patients [3]. Here, we asked whether LTG treatment would alter the behaviors of mice in a puzzle box test. The puzzle box test measured the latencies of mice to move from a bright start box to an enclosed goal box. The results of the 9 trials were analyzed with one-way ANOVA analysis and shown in Figure 4, which demonstrated the performance of groups for each trial (T1–T9). On the first day of testing, all mice had almost identical latencies to enter the goal box in each trial (T1–T3) (Figure 4(a)). On the second day of testing, APP/PS1 mice had increased latencies to enter the goal box compared to mice in other groups when the underpass was filled with bedding (burrowing puzzle), but LTG treatment significantly reduced the latencies (Figure 4(b)). Significant difference was also observed on T8 (plug puzzle) of third day between WT and APP/PS1 mice and reversed by the LTG treatment as well (Figure 4(c)). Accordingly, problem solving ability (T5 and T8) of APP/PS1 mice was significantly compromised and attenuated by LTG treatment (Figure 4(d)). Surprisingly, although a short-term memory deficit (T9, plug) was observed, LTG treatment was not able to reverse the deficit (Figure 4(e)). For the long-term memory deficiency, there was significant deterioration on the T7 (burrow) and could be reversed by the LTG treatment (Figure 4(f)). These results suggested that both executive and memory function were impaired in the 9-month-old AD mice and LTG treatment can prevent the executive dysfunction and long-term memory impairment but not the short-term memory deterioration in these mice.

## 4. Discussion

AD is neurodegenerative disease associated with significant declines in cognitive and behavioral functioning and is a leading cause of disability among elderly persons [22]. Executive dysfunction is reported in preclinical AD, even in the very early stage of disease, and negatively affects activities of daily living of patients [23]. The deteriorated executive function significantly compromises life quality of AD patients [2]. However, the current treatment for this clinical feature remains unsatisfactory so far. Antidepressants have been used in several clinical trials to treat executive dysfunction in different scenarios of disease with promising results [24], but the overall results are inconclusive [25].

The causes and pathophysiology of AD also remain unclear. Glial cell activation and progressive inflammatory response may be responsible for many features of AD. Glial cells are the major producers of inflammatory mediators and result in impaired neuronal function in AD. On the contrary, glial cell activation is enhanced under proinflammatory conditions [26]. Previous studies have suggested that IL-6 and IL-1*β* are overexpressed in AD brain, and this overexpression is directly associated with plaque formation, progression of dystrophic neurites, and neuronal overexpression of acetylcholinesterase in AD patients [13]. In this study, we also found high expression level of IL-6 and IL-1*β* in APP/PS1 mice and cultured astrocytes isolated from the APP/PS1 mice (Figures 1 and 2). LTG treatment can effectively inhibit overexpression of these two cytokines in AD mice. The literatures have well established that inflammation is responsible for the deterioration of executive function in patients [27, 28]. Therefore, we tested whether LTG treatment could simultaneously attenuate the executive dysfunction in the APP/PS1 mice. As anticipated, LTG significantly improved the behavioral performance of problem solving of the mice and long-term memory impairment in a puzzle box test (Figure 4). Interestingly, LTG could not prevent short-term memory deficit in the puzzle box test (Figure 4), which is inconsistent with a previous report [8]. When compared to other memory-relevant tests, mice in the puzzle box test are challenged with increasing difficulties and have to adopt different behaviors when facing each new problem. Therefore, mice need to translate a goal-directed intention into motor behavior for each individual test [17]. LTG may impede goal-directed motivation of mice due to its common adverse sedative effect. Further studies are needed in order to test if a lower dose of LTG can achieve the beneficial effects on not only executive but also memory function in AD mice.

It has been known that the action mechanism of LTG is related to an effect on voltage-gated sodium channels [29]. Despite their lack of electrical excitability, astrocytes can express voltage-activated Na+ channels with properties similar to the Na+ channels in neurons [30]. More interestingly, evidences also suggest that voltage-gated sodium channels play an important role in the inflammatory pain [31]. Our data here implied that LTG may inhibit the expression of proinflammatory cytokines, such as IL-1*β* and IL-6 in astrocytes, and prevent behavioral abnormalities.

High levels of cytokines have been detected in CSF and around A*β* plaques. Furthermore, it is well established that neuroinflammation play an important role in the pathogenesis of AD [32]. Cytokines, including IL-1*β* and IL-6, have been found to be extensively related to the cognitive dysfunction in animal models and patients [33]. More importantly, reduction of IL-1*β* and IL-6 is associated with better cognitive performances in AD mice [34]. The suppression effect of LTG on IL-1*β* and IL-6 in APP/PS1 mice further confirmed the key role of cytokines in deterioration of cognition of AD and also revealed mechanism of action by which LTG exerted the therapeutic effects on AD.

Although there is encouraging progress on the understanding of pathophysiology of AD during the past decades, executive dysfunction is still an unsolved major concern for the medical community and AD patients. For the first time, we demonstrated that LTG could exert beneficial effects on the executive performance in an AD mouse model by improving problem solving capability in a puzzle box test. Since LTG is an available prescription medication with relative safety profile, this finding may open a promising avenue to prevent executive function deterioration in AD.

## 5. Conclusion

APP/PS1 mice demonstrate obvious impairment of executive function and increased IL-1*β* protein level along with astrocyte activation. LTG can attenuate the executive dysfunction in the AD mice, while inhibiting astrocyte activation and production of cytokines. Early LTG treatment may be a promising way to prevent the progression of executive deterioration in AD.

## Supplementary Material

Animals were weighed every other week to monitor body weight changes. We found LTG could significantly prevent the loss of body weigh gain in APP/PS1 mice of 8 and 9 months old.
