# Supplementary material for: Lamotrigine Reduces Inflammatory Response and Ameliorates Executive Function Deterioration in an Alzheimer's-Like Mouse Model
Source: Biomed Res Int. 2016 Nov 30;2016:7810196. doi: 10.1155/2016/7810196 (PMC5155079; doi:10.1155/2016/7810196)
Supplement: Supplementary file 1 — Animals were weighed every other week to monitor body weight changes. We found LTG could significantly prevent the loss of body weigh gain in APP/PS1 mice of 8 and 9 months old. [file 7810196.f1.pdf]

Supplementary Fig. 1

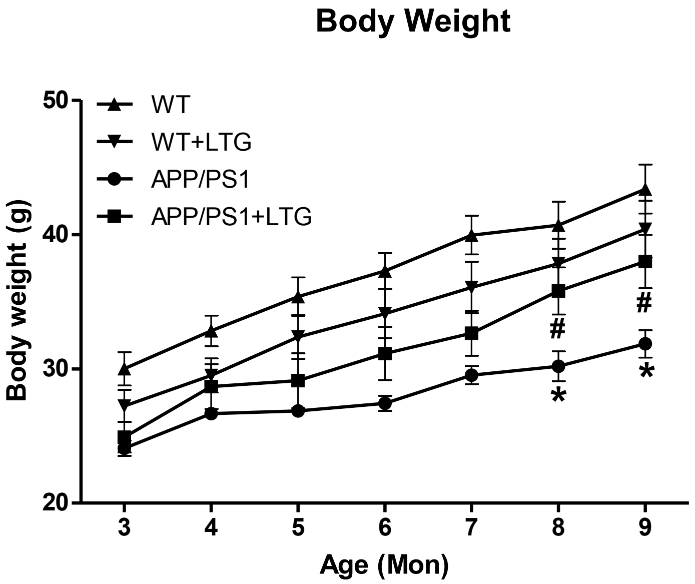

The body weight of each group were measured bi-weekly and analyzed here monthly. A significant lower weight gain was observed in the 8- and 9- month old APP/PS1 mice ($p<0.01$ vs WT) but LTG treatment (APP/PS1+LTG) could result in an increase of body weight gain in the APP/PS1 mice (($p<0.01$ vs APP/PS1). n = 7–15 in each group

Supplementary Fig. 2

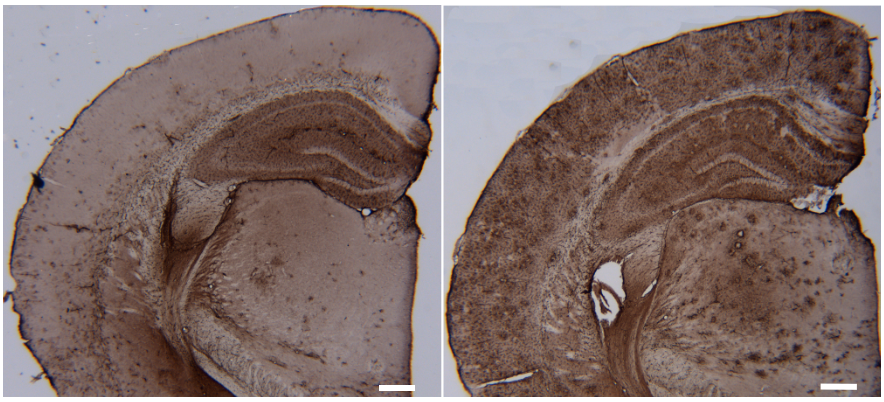

WT **APP/PS1**

GFAP staining in WT and APP/PS1 mouse brain section. Scale bar =500 μm

Supplementary Fig. 3

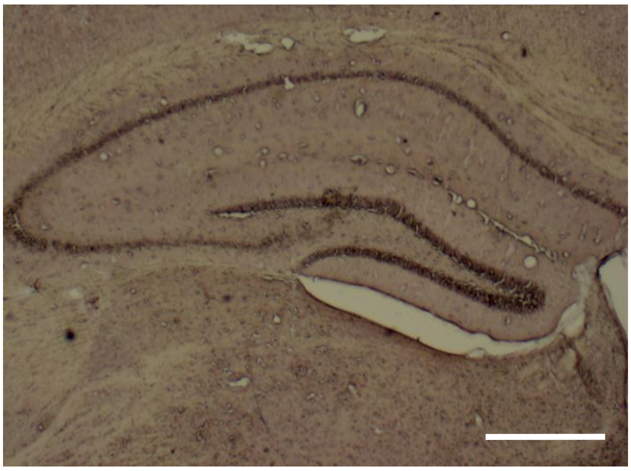

WT

Congo Red staining in WT mouse brain section. Scale bar =500 μm
